# Complete Genome Sequences of Mycobacterium chimaera Strains 850 and 852, Isolated from Heater-Cooler Unit Water

**DOI:** 10.1128/mra.01021-21

**Published:** 2022-01-20

**Authors:** David Pinzauti, Stefano De Giorgi, Valeria Fox, Elisa Lazzeri, Gabriele Messina, Francesco Santoro, Francesco Iannelli, Susanna Ricci, Gianni Pozzi

**Affiliations:** a Department of Medical Biotechnologies, University of Siena, Siena, Italy; b Department of Molecular and Development Medicine, University of Siena, Siena, Italy; c Programma Interdipartimentale Diagnostica Microbiologica, Sorveglianza e Caratterizzazione Patogeni Nosocomiali, Azienda Ospedaliera Universitaria Senese, Siena, Italy; Loyola University Chicago

## Abstract

The whole-genome sequences of Mycobacterium chimaera strains 850 and 852, which were isolated from two different water samples obtained from a heater-cooler unit at Siena University Hospital (Italy), were determined by combining Nanopore and Illumina technologies. Genomes of both strains 850 and 852 consist of a circular chromosome and five plasmids, with sizes of 6,275,686 bp and 6,453,144 bp, respectively.

## ANNOUNCEMENT

**FIG 1:**
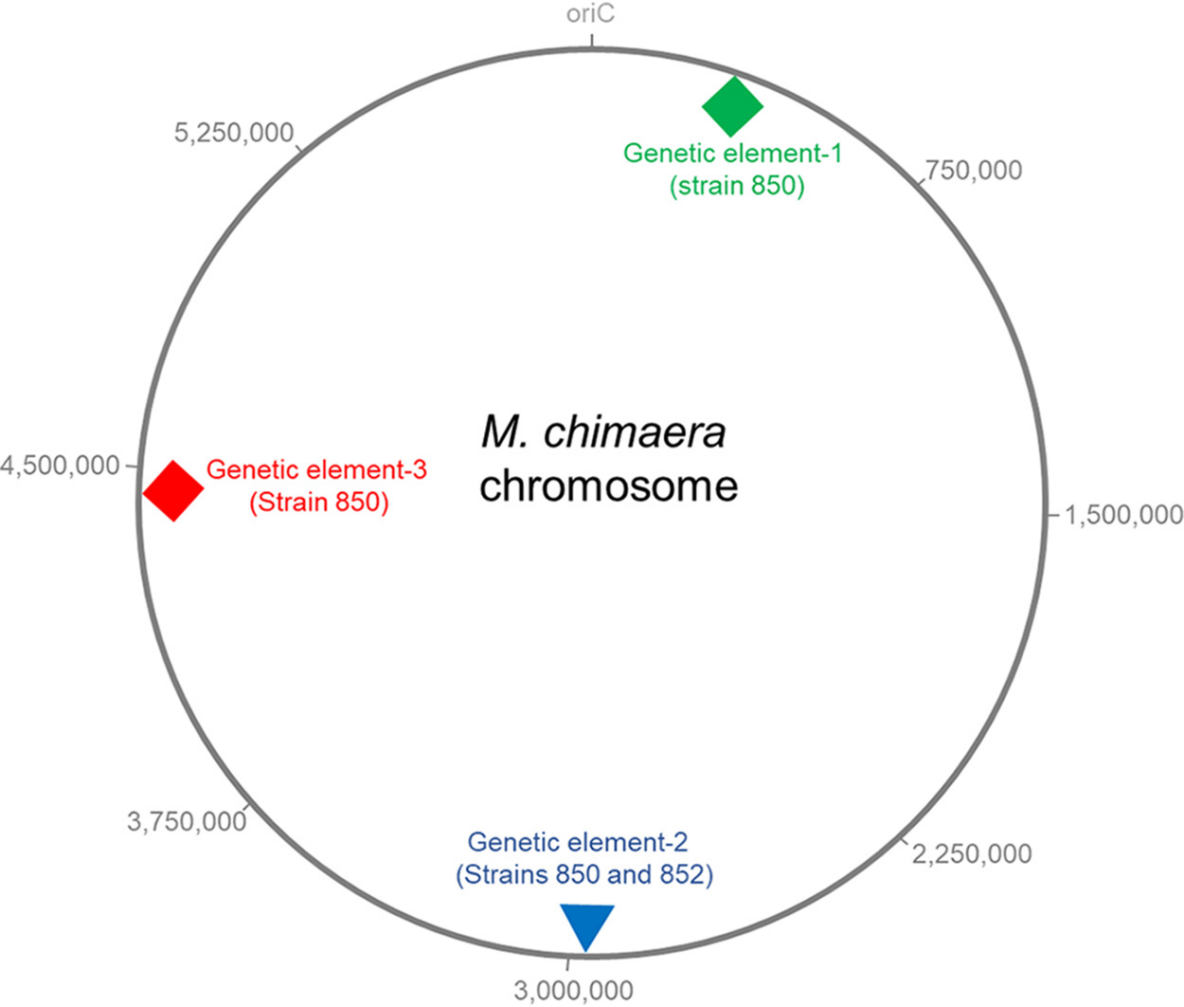
M. chimaera chromosome. Strains 850 and 852 have a common conserved chromosome backbone (indicated by the circle). The 147-kb genetic element 1 (green diamond) inserts at position 272509 of the strain 850 chromosome. The element carries a 94-kb operon putatively involved in degradation of polycyclic aromatic hydrocarbons and is present in 4 of 9 complete M. chimaera genomes. An additional 55-kb genetic element 2 (red diamond) inserts at position 4432414 and is present in 7 complete genomes. Both strains harbor a 10-kb genetic element (inverted blue triangle) integrated into an integration hot spot. Insertion occurred at position 2985490 in strain 850 and at position 2839393 in strain 852. Nucleotide positions on the map refer to the common backbone. The origin of replication (*oriC*) is reported.

Mycobacterium chimaera is a nontuberculous Mycobacterium species belonging to the Mycobacterium avium complex (MAC) (1–3). Recently, M. chimaera infections in cardiothoracic surgery patients were linked to the use of heater-cooler units (HCUs) (4–6). We sequenced the complete genomes of M. chimaera strains 850 and 852, which were isolated from two different filtered water samples obtained from a 3T HCU (Sorin, Italy) at Siena Hospital (Italy) in 2017, combining Illumina and Nanopore technologies. Strains were grown at 37°C on solid 7H11 medium (Becton Dickinson) for 5 days. Colonies were scraped and resuspended in 7H9 medium. DNA extraction was performed as described (7). Briefly, clumps were disrupted by vortex-mixing for 1 min with 20 glass beads (diameter, 3 mm; Sigma), and bacteria were inactivated for 3 h at 85°C. Samples were centrifuged (4,000 × *g* for 20 min), resuspended in GTE buffer (50 mM glucose, 25 mM Tris-HCl [pH 8], 10 mM EDTA) with 10 mg/mL lysozyme and 0.4 g glass beads (diameter, 150 to 212 μm; Sigma), vortex-mixed for 2 min, and incubated for 1 h at 37°C. Cells were lysed for 1 h at 55°C in 2% SDS with 1 mg/mL proteinase K. After the addition of NaCl and cetyltrimethylammonium bromide (CTAB), the solution was incubated for 10 min at 65°C, and DNA was purified twice with an equal volume of chloroform/isoamyl alcohol (24:1 [vol/vol]), precipitated in 2 volumes of ethanol, and resuspended in saline. Three micrograms of genomic DNA were size selected using 0.5× AMPure XP beads (Beckman Coulter). Nanopore sequencing libraries were prepared using the SQK-LSK108 kit following the manufacturer’s instructions (Oxford Nanopore Technologies [ONT]). Sequencing runs were managed by a GridION X5 device, enabling Guppy v5.0.12 (ONT) live base calling (high accuracy mode; quality score threshold, >9). Sequencing results were analyzed with NanoPlot v1.38.0 (8). Illumina sequencing was performed at MicrobesNG (University of Birmingham) using the Nextera XT library preparation kit, followed by paired-end sequencing (2 × 250 bp) on an Illumina HiSeq 2500 system. Nanopore reads were filtered using Filtlong (v0.2.0) (https://github.com/rrwick/Filtlong) to exclude reads smaller than 1,000 bases (--min_length 1,000) and the worst 5% of reads (--keep_percent 95). Illumina reads were trimmed using Trimmomatic v0.30 (9). Genomes were *de novo* assembled with Flye v2.8.3 (10) and polished with Medaka v1.4.1 (https://github.com/nanoporetech/medaka). A final Illumina-based polishing was performed with Pilon v1.2.4 (11). The genome of M. chimaera 852 was manually closed using Mauve v2.4.0 (12) and Bandage v0.8.1 (13) tools. Assembly quality was evaluated using Ideel (https://github.com/mw55309/ideel), and genomes were annotated with NCBI Prokaryotic Genome Annotation Pipeline (PGAP) v5.3 (14). Tools were run using default parameters unless otherwise specified. The 6,275,686-bp genome of M. chimaera 850 consists of a 6,076,815-bp circular chromosome and five plasmids, while the 6,453,144-bp genome of strain 852 consists of a 5,971,317-bp circular chromosome and five plasmids (Table 1). The 322-kb plasmid 1 of strain 852 contains 29 additional tRNA genes, which were not correctly resolved by the automated annotation (15). Comparison between the genomes, as performed by BLAST analysis, showed (i) a highly conserved chromosome backbone, (ii) two genetic elements integrated into the strain 850 chromosome, including the 147-kb genetic element 1 containing a 94-kb operon putatively involved in degradation of polycyclic aromatic hydrocarbons (Fig. 1), (iii) a 10-kb genetic element 2 integrated in a hot spot region of both strains, (iv) a conserved (100% identity) plasmid 4 in both strains (Table 1), and (v) the 95,688-bp sequence of strain 852 plasmid 2 contained in plasmid 1 of strain 850 and the 13,457-bp sequence of strain 850 plasmid 5 contained in plasmid 3 of strain 852.

**TABLE 1:** Genome features, data availability, and sequencing output for M. chimaera strains 850 and 852

Strain and parameter	Length (nucleotides)	No. of coding sequences	GC content (%)	No. of tRNAs	GenBank accession no.	No. of reads	No. of nucleotides	Avg length (nucleotides)	Coverage (×)	SRA accession no.
Mycobacterium chimaera 850										
Genome features										
Chromosome	6,076,815	5,622	67.6	47	CP084592					
Plasmid 1	97,267	101	65.98		CP084593					
Plasmid 2	39,887	34	65.0		CP084594					
Plasmid 3	32,137	30	64.85		CP084595					
Plasmid 4	21,123	20	65.11		CP084596					
Plasmid 5	13,457	17	65.77		CP084597					
Whole genome	6,275,686	5,824	67.53	47						
Sequencing output										
Illumina						1,572,298	335,350,687	213	53.44	SRR16127351
Nanopore						107,279	476,310,676	4,440	75.90	SRR16127352
Mycobacterium chimaera 852										
Genome features										
Chromosome	5,971,317	5,894	67.73	47	CP084586					
Plasmid 1	322,178	415	64.57	29	CP084587					
Plasmid 2	95,688	98	65.94		CP084588					
Plasmid 3	26,921	35	65.77		CP084589					
Plasmid 4	21,123	20	65.11		CP084590					
Plasmid 5	15,917	19	65.26		CP084591					
Whole genome	6,453,144	6,481	67.52	76						
Sequencing output										
Illumina						974,771	213,336,547	219	33.06	SRR16127349
Nanopore						180,586	737,333,218	4,083	114.26	SRR16127350

### Data availability.

All of the accession numbers for the genome sequences, sequencing projects, and raw reads are reported in Table 1. Details on the manual curation of the strain 852 assembly are available at https://zenodo.org/record/5722619#.Ycp-hGjMLrc.
